# Oral Alendronate Treatment for Severe Polyostotic Fibrous Dysplasia due to McCune-Albright Syndrome in a Child: A Case Report

**DOI:** 10.1155/2010/432060

**Published:** 2010-09-21

**Authors:** Ana Luiza Andrade Aragão, Ivani Novato Silva

**Affiliations:** 1 Divisão de Endocrinologia Pediátrica, Departamento de Pediatria, Hospital das Clínicas, Faculdade de Medicina, Universidade Federal de Minas Gerais (UFMG), Avenida Alfredo Balena, 190, sala 267, 30130-100 Belo Horizonte, MG, Brazil

## Abstract

Polyostotic fibrous dysplasia (FD) associated to McCune-Albright Syndrome (MAS) often leads to fractures, deformities, and bone pain resulting in bad quality of life. Parenteral bisphosphonates have been used in children and adolescents to improve these symptoms with few adverse effects. We evaluated the response to oral Alendronate in a girl with severe MAS FD and observed improved quality of life with reduction of bone pain.

## 1. Introduction

McCune-Albright Syndrome (MAS) is a rare disease presenting with the classical triad of polyostotic fibrous dysplasia (FD), skin hyperpigmentation (café-au-lait spots) and endocrine dysfunction, usually seen as precocious puberty in females. The mosaicism of mutations in the *GNAS1* gene results in hyperactive Gs*α* protein which causes activation of hormonal receptors and bone dysplasia [1–3]. There is increased osteoclastic activity, which can be detected by the elevation of biochemical markers of bone turnover, and normal bone marrow architecture is progressively replaced by abnormal fibrous tissue [4, 5]. 

bisphosphonates, antiresorption agents, largely used in the treatment of osteoporosis have been tried as an option for improvement of FD symptoms in MAS [6–9]. Most patients are treated with intravenous pamidronate, in a hospital regimen, or as outpatients in clinic settings. Alendronate (ALN) is a second-generation bisphosphonate that has, as an advantage, oral administration. It has been used for treatment of several disorders related to increased bone resorption; however, it is rarely used by the pediatric population. 

Since we could not find reports on the use of oral ALN in children with FD of MAS, although we are aware of an ongoing, as yet unpublished, study with children [4], we present the case of a girl with MAS treated during a one-year period with oral ALN.

## 2. Case Report

A 10.5-year-old white girl was admitted with complaints of severe weekly headache, diplopia, and nasal obstruction. She also had daily bone leg pains which caused frequent school absenteeism. She was in outpatient followup since she was 20 months old when it was noticed café-au-lait spots. By the age of 3 years she presented with left exophthalmos, and a severe form of FD of skull, which leads to the diagnosis of MAS. She had already undergone left optical nerve surgical decompression at 5 years of age and a second procedure was under consideration. At 6 and 10 years of age she was submitted to two turbinectomy procedures; however, she still had complaints of nasal obstruction. She did not present endocrinopathies or bone fractures episodes. When she was 7 years old she was treated twice with intravenous Pamidronate, 1 mg/kg/d during 3 days in a 6 month interval, and had presented vomiting and headache during the infusion. In-between these two treatments she received 10 mg/day of oral ALN for 30 days which was well tolerated. At 9 years of age she presented pubertal development (B2 Tanner stage). 

She was born to nonconsanguineous parents (Wt: 3000 g; Ht: 48 cm). She had a healthy brother. Her father and two uncles had café-au-lait spots, and her mother had high blood pressure and anxiety disorder. 

Physical examination showed severe craniofacial deformity, left proptosis and large café-au-lait spots with irregular borders in the left scalp and sacral region. Her weight was 36 kg (+1 SD) and height was 136.2 cm (0 SD); Body mass index was normal (BMI: 19.73 Kg/m^2^; 75 centile); she had a BP of 90/60 mmHg and B3 P2 Tanner stage. She was very upset with the bone pain and impaired quality of life, especially regarding school absenteeism, and a limited trial with oral ALN was prescribed.

Two and a half months before the ALN trial she had undergone screening for endocrinopathies (FT4: 1.0 mg/dL (RV: 0.8–1.9), TSH: 1.35 mUI/mL (RV: 0.4–5.0), PRL: 2.2 ng/mL (RV: 1.9–25), Cortisol: 13.9 ng/dL (RV: 5.0–25)—RV: Reference value) and renal phosphate wasting, which was absent. GH levels after oral 75 g dextrose were normal. Serum calcium, phosphorus, 25–OH Vitamin D and PTH levels were normal (Table 1). Bone scintigraphy revealed typical pattern of polyostotic FD with intense uptake in facial bones, right femur, right arm and ribs. DEXA showed normal total body bone mineral density (BMD: 0.907 g/cm^2^; +0.09 SD). Computerized Tomography of skull showed fibrous dysplasia lesions without signs of increased intracranial pressure. Biochemical markers of bone turnover, alkaline phosphatase and 24-hour urinary hydroxyproline levels were elevated. Blood count, electrocardiogram and serum liver enzymes were normal.

**Table 1:** Biochemical markers of bone metabolism.

**Weeks of followup**	**PTH**	**25 OHD**	***Ionized* Ca**	**Total Ca**	**P**	**ALP**	**Urinary 24 h hydroxyproline**
0	39.2	79.4	NA	9.0	4.2	852	*112
8	NA	NA	1.25	9.3	NA	864	NA
29	57.7	NA	NA	8.5	3.9	798	*214
47	70.6	NA	NA	9.6	4.0	608	NA
61	61.7	NA	1.12	8.9	3.6	585	**9.4
RV	10–69 pg/mL	14–80 ng/mL	1.12–1.32 mmol/L	8.4–10.2 ng/dL	2.5–4.5 ng/dL	50–530 UI/L	*12–58 mg/24 h**70–140 mg/24 h

NA: not available; RV: reference value; Ca: calcium; P: phosphorus; ALP: serum alkaline phosphatase.

## 3. ALN Treatment

After the patient and her mother signed an informed consent form, ALN (70 mg tablets with 200 ml of water) was administered once a week, initially under medical supervision. She was advised to be fasting and remain upright for at least 30 minutes following ingestion of the drug. After two weeks, the patient continued medication at home, with no difficulties. ALN treatment was interrupted for 15 weeks (between 8 and 29 weeks of followup) due to a complaint of muscle pain and dysphagia during the postoperative period of her third turbinectomy. She also presented mild self-limited dyspepsia with no need for treatment interruption. 

Throughout her followup visits she repeatedly reported feeling better and not having missed any more classes. Improvement was also evaluated through a numeric pain scale where the perception of pain varied from 0 to 10. Pain perception went from an intensity of 8 to 5 following 7 months of treatment. After 10 months of treatment the pain intensity was 3 and after 12 months of treatment she felt no pain. She presented menarche 5 months after the beginning of the medication. Growth velocity was of 6 cm/y associated with proportional advancement of bone age during the 2 year study (CA: 11 y3 m and BA: 11 y). Sclerotic bands in the left wrist metaphyses were observed on plain X-ray, after treatment.

Improvement during ALN treatment was also attested by the lowering of urinary hydroxiproline levels during followup and near complete normalization of alkaline phosphatase (Table 1). 

She was treated with ALN for a year and the benefits lasted during the year following the end of treatment. At her last visit she was 14 years old; she was clinically stable and felt no pain. The results of a new screening for endocrinopathies did not show abnormalities.

## 4. Discussion

The primary and likely only effect of bisphosphonate treatment observed in MAS patients is the reduction or complete resolution of bone pain. Reduction of bone fractures, no harm to linear growth or altered fracture healing and better quality of life have been described [6, 10, 11]. Until now, the majority of pediatric patients received parenteral pamidronate [6, 11–16]; however, there is no consensus on the effectiveness of this treatment [7, 17]. 

Chapurlat et al. found that almost 50% of patients had radiological improvement of lesions as well as increase in cortical thickness and filling of lytic lesions [12]. 

Improvement of bone pain may be explained by the antiosteoclastic action of the drug, with reduction of cytokines and other pain-related substances released by the lesions. Furthermore, improvement of bone mineral density may occur due to inhibition of bone resorption leading to improvement of surgical outcome in MAS patients treated with bisphosphonates, yet a controversial issue [4, 14]. 

ALN is a biphosphonate which is largely used in the treatment of osteoporosis in adults [18] and osteogenesis imperfecta in children [10, 19]. It was related to satisfactory improvement of bone pain and less affected sites in the scintigraphy scan in some reports [8, 20]. 

ALN oral administration is considered to be safe and well tolerated. Most common adverse effects are upper gastrointestinal, mainly, esophageal irritation, which can be prevented with special care during the administration. Headache, diarrhea, intestinal constipation and atrial fibrillation may also occur [21–23]. Recently there were reports of esophageal cancer with oral bisphosphonate use in adult patients [24]. Sclerotic bands, observed in wrist metaphyses on plain X-ray, are often described in the literature and appear to have no clinical implications [25]. There is also some concern about longer-term possible side effects of bisphosphonates on future pregnancies of treated females [15].

Instead of classical daily administration, the new therapeutic regimens such as 70 mg/weekly dose given to adults has also been used in children with osteogenesis imperfecta (according to the weight) with good tolerance [10]. The 70 mg/weekly dose administered to this girl was based on treatment protocols that have been used in pediatric patients with different diagnoses [10, 19, 26]. These weekly regimens may improve compliance and reduce the incidence of adverse effects. 

Nonetheless, the decision to initiate bisphosphonates and the adequate time to cease their use is controversial. There are no well-defined criteria for precise indication and response to treatment. Different parameters for treatment suspension in children with osteoporosis have been reported: BMD Z Score  SD; reduction of 50% in fracture incidence; resolution of bone pain (the most useful indicator of treatment effect) or occurrence of adverse effects [19, 21, 27]. 

There is no consensus on the changes in the markers of bone turnover during bisphosphonate therapy. Alkaline phosphatase (bone formation marker) and urinary hydroxyproline (bone resorption marker) are indicators of severity and activity of osseous lesions in MAS [4]. Lowering of the biochemical markers of bone resorption is more pronounced than the changes in formation markers after bisphosphonate treatment [21, 22, 27–29]. There are reports of significant reduction in alkaline phosphatase, osteocalcin, urinary hydroxyproline and urinary NTX during bisphosphonate use [12]. However, reference values for some of these biochemical markers are not well established in the pediatric population mainly because their increment could be a reflection of the normal bone growth and remodeling processes [1]. Although we noticed a trend towards normality in our patient biochemical markers it is not possible to foresee if there will be a sustained beneficial effect to treatment. There are reports of improvement in BMD persisting for 2 years after interruption of intravenous bisphosphonate treatment in children with osteogenesis imperfecta [19]; biochemical markers of bone turnover were normal after 6–9 months of treatment [27]. 

Until standard criteria for treatment interruption have been established we believe the endpoint should be clinical improvement. Improvement of bone pain and of daily activities including reduction in school absenteeism, showing an increase in quality of life were the criteria which defined interruption of treatment in the present paper. 

We considered the outcome to have been favorable although it is still not possible to predict its endurance or reproducibility*.*

We concluded that weekly ALN regimen was a good option for improving symptoms of FD of MAS in this girl, due to ease of administration and lack of severe adverse effects reported in the patient. We also believe there is an urgent need for ongoing studies addressing the many unanswered questions regarding the use of bisphosphonates in pediatric patients, in order to safely use this medication.

## References

[B1] Völkl TMK Dörr HG McCune-Albright syndrome: clinical picture and natural history in children and adolescents Journal of Pediatric Endocrinology and Metabolism 2006 19 2 551 559 10.1515/JPEM.2006.19.S2.551 16789617

[B2] Spiegel AM Komrower lecture: inborn errors of signal transduction: mutations in G proteins and G protein-coupled receptors as a cause of disease Journal of Inherited Metabolic Disease 1997 20 2 113 121 10.1023/A:1005393501786 9211183

[B3] Defilippi C Chiappetta D Marzari D Mussa A Lala R Image diagnosis in McCune-Albright syndrome Journal of Pediatric Endocrinology and Metabolism 2006 19 2 561 570 10.1515/JPEM.2006.19.S2.561 16789618

[B4] Diaz A Danon M Crawford J McCune-Albright syndrome and disorders due to activating mutations of GNAS1 Journal of Pediatric Endocrinology and Metabolism 2007 20 8 853 880 10.1515/JPEM.2007.20.8.853 17937059

[B5] DiCaprio MR Enneking WF Fibrous dysplasia: pathophysiology, evaluation, and treatment Journal of Bone and Joint Surgery A 2005 87 8 1848 1863 10.2106/JBJS.D.02942 16085630

[B6] Lala R Matarazzo P Andreo M Marzari D Bellone J Corrias A de Sanctis C Bisphosphonate treatment of bone fibrous dysplasia in McCune-Albright syndrome Journal of Pediatric Endocrinology and Metabolism 2006 19 2 583 593 10.1515/JPEM.2006.19.S2.583 16789621

[B7] Lane JM Khan SN O'Connor WJ Nydick M Hommen JP Schneider R Tomin E Brand J Curtin J Bisphosphonate therapy in fibrous dysplasia Clinical Orthopaedics and Related Research 2001 382 6 12 10.1097/00003086-200101000-00003 11154006

[B8] Kitagawa Y Tamai K Ito H Oral alendronate treatment for polyostotic fibrous dysplasia: a case report Journal of Orthopaedic Science 2004 9 5 521 525 10.1007/s00776-004-0809-0 15449129

[B9] Chao K Katznelson L Use of high-dose oral bisphosphonate therapy for symptomatic fibrous dysplasia of the skull: case report Journal of Neurosurgery 2008 109 5 889 892 10.3171/JNS/2008/109/11/0889 18976079

[B10] Ward LM Denker AE Porras A Shugarts S Kline W Travers R Mao C Rauch F Maes A Larson P Deutsch P Glorieux FH Single-dose pharmacokinetics and tolerability of alendronate 35- and 70-milligram tablets in children and adolescents with osteogenesis imperfecta type I Journal of Clinical Endocrinology and Metabolism 2005 90 7 4051 4056 10.1210/jc.2004-2054 15827104

[B11] Isaia GC Lala R Defilippi C Matarazzo P Andreo M Roggia C Priolo G De Sanctis C Bone turnover in children and adolescents with Mccune-Albright syndrome treated with pamidronate for bone fibrous dysplasia Calcified Tissue International 2002 71 2 121 128 10.1007/s00223-001-1098-7 12200645

[B12] Chapurlat RD Hugueny P Delmas PD Meunier PJ Treatment of fibrous dysplasia of bone with intravenous pamidronate: long-term effectiveness and evaluation of predictors of response to treatment Bone 2004 35 1 235 242 10.1016/j.bone.2004.03.004 15207763

[B13] Plotkin H Rauch F Zeitlin L Munns C Travers R Glorieux FH Effect of Pamidronate treatment in children with polyostotic fibrous dysplasia of bone Journal of Clinical Endocrinology and Metabolism 2003 88 10 4569 4575 10.1210/jc.2003-030050 14557424

[B14] Zacharin M O'Sullivan M Intravenous pamidronate treatment of polyostotic fibrous dysplasia associated with the McCune Albright syndrome Journal of Pediatrics 2000 137 3 403 409 10.1067/mpd.2000.107836 10969268

[B15] Shaw NJ Bishop NJ Bisphosphonate treatment of bone disease Archives of Disease in Childhood 2005 90 5 494 499 10.1136/adc.2003.036590 15851432 PMC1720410

[B16] Bolger WE Ross AT McCune-Albright syndrome: a case report and review of the literature International Journal of Pediatric Otorhinolaryngology 2002 65 1 69 74 10.1016/S0165-5876(02)00125-8 12127226

[B17] DiMeglio LA Bisphosphonate therapy for fibrous dysplasia Pediatric Endocrinology Reviews 2007 4 4 440 445 17982393

[B18] Unal E Abaci A Bober E Büyükgebiz A Efficacy and safety of oral alendronate treatment in children and adolescents with osteoporosis Journal of Pediatric Endocrinology and Metabolism 2006 19 4 523 528 16759038

[B19] Bachrach LK Ward LM Clinical review: bisphosphonate use in childhood osteoporosis Journal of Clinical Endocrinology and Metabolism 2009 94 2 400 409 10.1210/jc.2008-1531 19033370

[B20] Yamamoto T Ozono K Shima M Yoshikawa H Okada S Alendronate and pharmacological doses of 1*α* OHD3 therapy in a patient with McCune-Albright syndrome and accompanying hypophosphatemia Journal of Bone and Mineral Metabolism 2002 20 3 170 173 10.1007/s007740200024 11984700

[B21] Srivastava T Alon US The role of bisphosphonates in diseases of childhood European Journal of Pediatrics 2003 162 11 735 751 10.1007/s00431-003-1298-4 14523647

[B22] Biswas PN Wilton LV Shakir SAW Pharmacovigilance study of alendronate in England Osteoporosis International 2003 14 6 507 514 10.1007/s00198-003-1399-y 12730757

[B23] Solomon DH Rekedal L Cadarette SM Osteoporosis treatments and adverse events Current Opinion in Rheumatology 2009 21 4 363 368 10.1097/BOR.0b013e32832ca433 19412101

[B24] Wysowski DK Reports of esophageal cancer with oral bisphosphonate use The New England Journal of Medicine 2009 360 1 89 90 10.1056/NEJMc0808738 19118315

[B25] Grissom LE Harcke HT Radiographic features of bisphosphonate therapy in pediatric patients Pediatric Radiology 2003 33 4 226 229 12709749 10.1007/s00247-003-0865-1

[B26] Bianchi ML Cimaz R Bardare M Zulian F Lepore L Boncompagni A Galbiati E Corona F Luisetto G Giuntini D Picco P Brandi ML Falcini F Efficacy and safety of alendronate for the treatment of osteoporosis in diffuse connective tissue diseases in children: a prospective multicenter study Arthritis and Rheumatism 2000 43 9 1960 1966 10.1002/1529-0131(200009)43:9<1960::AID-ANR6>3.0.CO;2-J 11014345

[B27] Waterhouse KM Auron A Srivastava T Haney C Alon US Sustained beneficial effect of intravenous bisphosphonates after their discontinuation in children Pediatric Nephrology 2007 22 2 282 287 10.1007/s00467-006-0306-0 17033813

[B28] Srivastava AK MacFarlane G Srivastava VP Mohan S Baylink DJ A new monoclonal antibody ELISA for detection and characterization of C-telopeptide fragments of type I collagen in urine Calcified Tissue International 2001 69 6 327 336 10.1007/s00223-001-1034-x 11800229

[B29] Pedrazzoni M Alfano FS Gatti C Fantuzzi M Girasole G Campanini C Basini G Passeri M Acute effects of bisphosphonates on new and traditional markers of bone resorption Calcified Tissue International 1995 57 1 25 29 10.1007/BF00298992 7671161

